# Effectiveness of Low-Level Laser Therapy during Tooth Movement: A Randomized Clinical Trial

**DOI:** 10.3390/ma12132187

**Published:** 2019-07-08

**Authors:** Gaetano Isola, Marco Matarese, Francesco Briguglio, Vincenzo Grassia, Giacomo Picciolo, Luca Fiorillo, Giovanni Matarese

**Affiliations:** 1 Department of General Surgery and Surgical-Medical Specialties, Via Plebiscito 628, University of Catania, 95124 Catania, Italy; 2 Department of Biomedical, Odontostomatological Sciences and Morphological and Functional Images, School of Dentistry, Via Consolare Valeria 1, University of Messina, 98125 Messina, Italy; 3 Multidisciplinary Department of Medical-Surgical and Dental Specialties, Second University of Naples, Via Luigi de Crecchio 6, 80138 Naples, Italy

**Keywords:** laser therapy, diode Laser, orthodontic tooth movement, pain, clinical trial

## Abstract

The present study evaluated the effects of low-level laser therapy (LLLT) by means of a diode laser in accelerating orthodontic tooth movement (OTM). After extraction of the first upper premolars for orthodontic purpose, 82 maxillary canines which needed distalization were analyzed in 41 enrolled patients (21 males, 20 females, mean age 13.4 ± 2.1 years). On all experimental sites, an orthodontic force of 50/N was applied by a nickel-titanium (NiTi) closed coil spring (G&H, Franklin, IN, USA) in order to obtain the space closure. Using a split mouth randomized design, the test side was treated using a diode laser (Wiser Laser Doctor Smile, Brendola, Italy) operating at 810 nm wavelength in continuous wave mode at both the buccal and palatal side on three points/side (distal, medial and mesial) (1 W output power, continuous wave of 66.7 J/cm^2^, energy density of 8 J) at baseline and at 3, 7, and 14 days and every 15 days until the space closed. On the control side, the opposite selected canine was treated only using orthodontic traction. The primary outcome chosen was the overall time needed to complete the levelling and closing space, measured on a study cast. The secondary outcome chosen was the evaluation of pain levels related to tooth traction, using a Visual Analogue Scale (VAS), recorded at 3, 7, and 14 days after treatments. The mean space closures of the maxillary canines were comparable between groups [Test, 4.56 mm (95% CI 3.9–4.8); Control, 4.49 mm (95% CI 3.8–4.7), *p* = 0.456]. The laser group yielded less mean time [84.35 ± 12.34 days (95% CI 79.3–86)] to accomplish space closure compared to the control group [97.49 ± 11.44 days (91.7–102.3)] (*p* < 0.001). The test side showed a significant reduction in the average range of dental pain at 3 [Test, 5.41 (95% CI 5.1–5.6); Control, 7.23 (95% CI 6.9–7.6), *p* < 0.001], 7 [Test, 4.12 (95% CI 3.8–4.7); Control, 5.79 (95% CI 5.4–5.8), *p* < 0.001], and at 14 days [Test, 2.31 (95% CI 1.8–2.3); Control, 3.84 (95% CI 3.3–4.2), *p* < 0.001] after treatment (*p* < 0.001). This study demonstrates that the use of LLLT therapy was effective in accelerating tooth movement and reducing pain levels related to OTM.

## 1. Introduction

Orthodontic tooth movement (OTM) is a complex process defined as an adaptive biologic response to interference in the physiologic equilibrium of the dentofacial structures exerted by an externally applied force [1]. As a result of the organized periodontal tissue remodeling after the application of mechanical forces, bone remodeling during OTM is a biologic mechanism that involves an acute inflammatory response and increment in pain perception by patients [2,3].

More specifically, during OTM, remodeling of the periodontium stimulates bone resorption contiguous to the periodontal ligament in the side of compression, bone deposition in the side of tension, and degeneration and reorganization of the periodontal ligament [4,5,6].

The initial tooth displacement due to orthodontic force determines, especially in the early stages of OTM [2,4], the increase of stress in the periodontal ligament which determines the osteogenic response of the alveolar bone responsible for the subsequent tissue remodeling [7,8,9]. This could result in a significant reduction in the speed of OTM and in related phenomena such as in gingivitis, dental ankyloses, and root resorption [10,11,12].

Therefore, alternative and minimally invasive therapies and methods have been developed in order to decrease orthodontic treatment time [9,11]. Mechanical vibration [13], corticotomy [14], piezocision [15], as well as several pharmacological adjuvants [16] have been proposed as possible methods for accelerating OTM. However, although most of these were effective, their use may not be clinically feasible due to their invasive nature or possible side effects [17].

Recently, low-level laser therapy (LLLT) has been shown to be effective in inducing remodeling processes in the soft and hard oral tissues, healing through its photobiostimulation effects [18,19].

The use of LLLT during OTM has been shown to be useful and effective in reducing orthodontic pain and in the photobiomodulation that might accelerate orthodontic tooth movement, as well as inhibiting the release of pain mediators related to analgesia [20,21]. The biostimulation on oral tissues induced by LLLT is also due to the cellular absorption of laser light by the target tissue which leads to the activation of intracellular signaling cascades, which increases cellular metabolism, and anti-inflammatory changes of soft and hard oral tissues [19]. This process has been shown to stimulate, in the long term, a better tooth movement and osteoclast formation on the compression side during experimental tooth movement, determining and enhancing the rate of OTM time [22,23].

Kawasaki and Shimizu [24] have shown that in vivo in rats, LLLT stimulated tooth movement and osteoclast formation on the compression side during experimental OTM. Some other studies [25,26] have demonstrated that low-level laser therapy accelerates tooth movement via the receptor activator of the nuclear factor kappa B (RANK), RANK ligand (RANKL), and macrophage-colony stimulating factor along with its receptor (c-fms).

The LLLT therapy can be performed by a wide range of lasers currently on the market including Nd: YAG, He-Ne and diode lasers [17,18]. Among these, the diode laser has been shown to be widely penetrating at tissue level with reduced light absorbed by the tissues [27]. The diode laser is highly penetrant and minimally absorbed by water [19]. In addition, diode lasers are inexpensive devices and easy to miniaturize, which has led to the development of a wide range of clinical applications. The actions exerted by diode laser have been demonstrated to accelerate tissue repair and to determine a relatively low mechanical stress to the roots of the teeth which are already subjected to orthodontic traction [28]. At high power, diode lasers have been shown to have a hemostatic, bactericidal and detoxifying effect [22]; Moreover, it has been demonstrated that diode laser can be used to perform incision and hemostasis of soft tissue and caries removal [29] whereas, at low power, they can be applied for periodontal therapy and treatment of hypersensitivity [22,30,31]. However, the effect of diode laser therapy during OTM, although its positive effects have been demonstrated in some experimental studies [22,28], has not yet been fully understood.

Therefore, the objective of the present study was to evaluate the effect of laser-induced biostimulation using a diode laser operating at 810 nm in order to accelerate OTM. Moreover, the secondary objective of the study was to evaluate the effectiveness of LLLT on the pain experienced by the patient during OTM. The null hypothesis to invalidate was that there were no differences between treatments regarding the speed of OTM and the pain experienced.

## 2. Materials and Methods 

### 2.1. Study Design

Patients, who needed orthodontic treatment were enrolled for the present randomized, split mouth clinical trial (RCT). The study was approved by the local ethics committee of the University of Messina (protocol 18/18). The report of this trial was performed following the CONSORT guidelines [32] (Supplementary Materials).

### 2.2. Study Sample

One hundred and twelve patients, matched for age and gender, who needed maxillary first premolar extraction and bilateral maxillary canine distalization for orthodontic reasons were selected from those who attended the School of Dentistry of the University of Messina, Messina, Italy from January 2018 to November 2018.

The inclusion criteria were: (1) Patients who required extraction of the first maxillary premolars due to excessive dental crowding or biprotrusion for orthodontic purposes; (2) presence of permanent dentition; (3) absence of any systemic condition that could have influenced the results of the study; (4) previous orthodontic treatment; (5) previous history of trauma; (6) past or present signs of periodontal disease.

**Figure 1:**
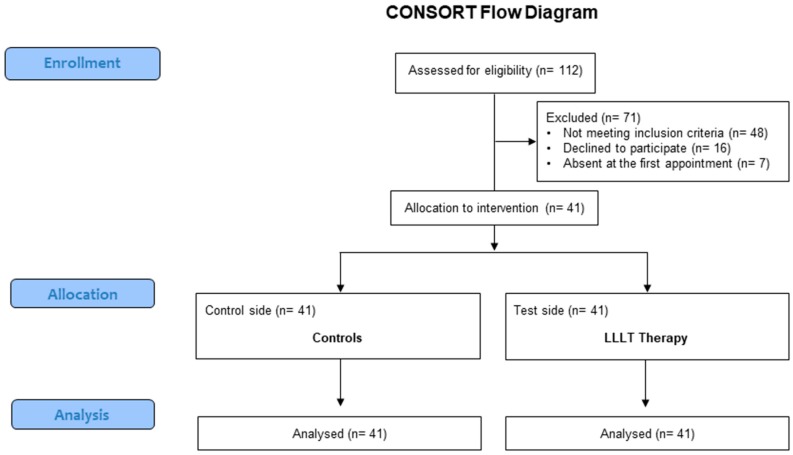
Flowchart of the study.

After a first screening, 71 patients were excluded from the final sample because they did not meet the inclusion criteria (n = 48), declined to participate (n = 16), or did not attend the first appointment (n = 7). Thus, for this study 41 patients, 21 males and 20 females, matched by age and sex (mean age 13.4 ± 2.1 years, range 10.2–18.4 years), were finally included in the present study (Figure 1).

### 2.3. Power Sample Analysis

The power sample was calculated with an effect size of 0.60, α = 0.050 and with a power level of 0.80 for tooth movement in mm, which was the primary variable chosen using a statistical software (G*Power, Heinrich-Heine-Universität Düsseldorf, Germany) [27,28,33]. The minimum sample calculated for analysis was estimated to be 37 teeth per group to be necessary. Considering expected dropouts, 41 elements per group were enrolled for the study.

### 2.4. Randomization

All patients were assigned, using a simple randomization technique, in a 1:1 allocation ratio. Randomization was carried out using a computerized random number generator. Each site was masked in sequentially sealed and numbered envelopes and allocated to receive one of the two treatments (test and control) using randomization in permuted blocks.

Then, each selected tooth was assigned to one of the two groups. Only one clinician, not involved in the subsequent phases of the trial (LF) performed the random allocation sequence and assignment to intervention.

For the study, a total of 82 maxillary canines were selected and evaluated, and divided into two groups, test (41 canines) and control side (41 canines). 

### 2.5. Treatment

All patients underwent a fixed orthodontic treatment with metal brackets 0.022–0.028 inch (Ormco Corp., Orange, CA, USA). After the initial alignment and levelling phase which required approximately 6 months, a final stainless-steel wire 0.016 × 0.022 stainless steel (ss) was placed.

**Figure 2:**
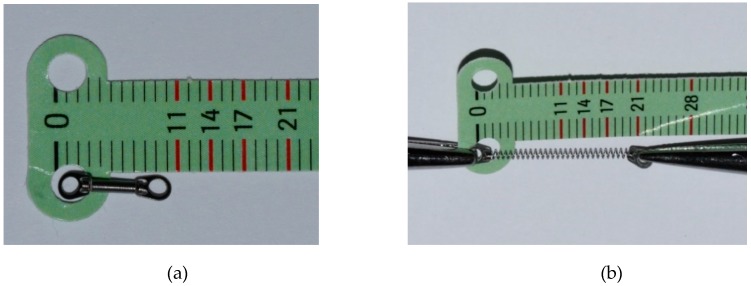
(**a**) The closed NiTi coil spring of 9 mm used for the study; (**b**) The NiTi coil spring activated with a force of 50/N.

Then, after 21 days, the extractions of the first premolars were performed. Subsequently, 7 days after premolar extractions, a segmented arch of 0.016−0.022 ss was applied in association with a closed nickel-titanium (NiTi) coil spring (9 mm in length, G&H Orthodontics Wire Company, Franklin, IN, USA) which delivered a force of 50/N at maximum extension (Figure 2). The coil spring was applied on the buccal side in order to obtain the space closure of the first premolars. The force exerted by the coil spring was measured by a commercial dynamometer (Morelli, São Paulo, Brazil). A subsequent and progressive reactivation of the spring was carried out every 21 days until a total closure of the extraction space was obtained.

The canine of the control side was subjected to distalization by using the coil spring only.

On the test side, the LLLT on the canine was carried out using a diode laser operating at 980 nm (Wiser Laser Doctor Smile, Lambda, Brendola, Italy) with a wavelength of 810 nm (1 W of output power, a continuous wave of 66.7 J/cm^2^) and 0.6 mm optic with fiber.

The laser device was applied, with the laser tip in contact with gingival tissues, to both the buccal and palatal side starting from the center of the root on 3 points/side (distal, medial and mesial). Each area was irradiated for 15 s, with an energy density of 8 J (2 × 40 × 100 mW) in total. Protective eyewear was used to protect patients and operators throughout the irradiation time.

Irradiation was performed following activation of the NiTi spring on the maxillary canines and repeated at baseline and after 3, 7, and 14 days after the first application and every 15 days until levelling and closure of the tooth extraction space was achieved. During this phase, the patient was not blinded to treatment (e.g. test or control).

After irradiation, the measurement of the dental displacement was carried out, by the same examiner (GI) on both the test and control sides using a digital caliper (Mitutoyo, Kawasaki, Japan) after the first premolar extraction (T0), after 1-month treatment commencement (T1), after 2 months (T2), and at the end of the leveling and closing stage (T3). 

### 2.6. Outcomes

The primary outcome was the overall time needed to complete alignment and space closure of the maxillary canines, measured in millimeters. This distance was evaluated on the study cast, by the same examiner (GM), at four time points: at baseline, after the first premolar extraction (T0), after 1-month from treatment commencement (T1), after 2 months (T2), and at the end of the leveling and closing stage (T3). Little’s irregularity index (LII) was chosen to measure the change on the casts using a digital caliper (Mitutoyo, Japan). To calculate outcome measures, a maxillary alginate impression was taken to make study casts at four time points: before insertion of the first archwire (T0), and at each time point (T1, T2, and T3).

The secondary outcome was the pain experienced by the patient during tooth movement. At baseline, each patient was asked to record the pain using a visual analogue scale (VAS) [34]. 

The patients were carefully instructed regarding how to complete VAS for both left and right sides. Patients were asked to fill their VAS score at 3, 7, and 14 days after treatment. A score of 0 represented the absence of any pain/discomfort while a score of 10 intended any pain considered to be intolerable.

At 6 months after therapy, each patient was recalled by the same examiner (GI), for a final check-up with a clinical and radiological analysis (using Rinn periapical Rx) for the evaluation of possible damage to soft and hard tissues or root reabsorption of the treated tooth. At this stage, every possible complication at the site level was recorded during a clinical examination.

The inter-examiner reliability test was performed and resulted in an agreement of 87.1% (k = 0.71) for the primary outcome chosen. The intra-examiner agreement was evaluated by Cohen’s k coefficient, resulting in 0.829, and predicted a good degree of reliability. The kappa coefficient was also calculated for the measurements taken at each follow-up session, and an acceptable degree of reliability (ICC = 0.769) was established for every examination. To assess measurement reliability, at T1, 12 dental casts were randomly chosen, and LII measurements were repeated.

### 2.7. Statistical Analysis

All the numerical data were expressed by mean values ± Standard Deviation (SD) while the categorical variables were expressed as number and percentage. The data were normally distributed as verified by the Kolmogorov–Smirnov test; consequently, a parametric approach was used. The data were compared by two-sample t-tests. The ANOVA test was used for the analysis of differences in post-therapy pain levels (T1, T2, T3). All data were processed using SPSS software (IBM, Milano, Italy), version 11.0 for Windows. A *p* value < 0.05 was set as statistically significant.

## 3. Results

All participants successfully completed the study. Table 1 shows the average speed of tooth movement to accomplish space closure for the test side (diode laser) and the control side. The null hypothesis was invalidated.

### 3.1. Primary Outcome

The mean space closures of the maxillary canines in millimeters were comparable between groups [Test, 4.56 mm (95% CI 3.9–4.8); Control, 4.49 mm (95% CI 3.8–4.7), *p* = 0.456]. The laser group yielded less mean time (84.35 ± 12.34 days) to accomplish space closure compared to the control group (97.49 ± 11.44 days), with a mean reduction in the overall treatment time for the test side compared to the control side (*p* < 0.001) (Table 1).

The mean percentage of the days of levelling and alignment improvement was significantly higher in the test side than in the control side at T1 and T2, while there was no statistical significance between groups at T3 (*p* = 0.878). At T1, the test side presented a higher percentage of levelling and alignment improvement percentage (65.36 ± 11.39%) compared to the control side (44.39 ± 15.51%, *p* = 0.003). Similarly, at T2, the test side showed a higher percentage of levelling and alignment improvement (89.42 ± 7.16%) compared to the control side (68.66 ± 15.12%, *p* < 0.001) (Table 2).

### 3.2. Secondary Outcome

**Figure 3:**
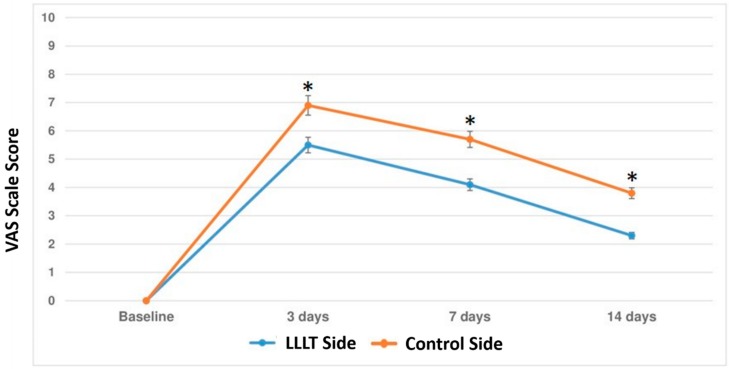
The results of the pain levels experienced in diode laser and control groups at each follow-up session (3, 7, and 14 days). Error bars represent the Standard Deviation (±SD). *, *p* < 0.001, comparison between groups at each follow-up session.

Figure 3 shows the average levels of the VAS score at the different follow-up sessions on the test and control side. The test side (diode laser) showed a significant reduction in the average range of dental pain due to orthodontic traction at 3 [Test, 5.41 (95% CI 5.1–5.6); Control, 7.23 (95% CI 6.9–7.6), *p* < 0.001], 7 [Test, 4.12 (95% CI 3.8–4.7); Control, 5.79 (95% CI 5.4–5.8), *p* < 0.001] and at 14 days [Test, 2.31 (95% CI 1.8–2.3); Control, 3.84 (95% CI 3.3–4.2), *p* < 0.001] after first laser treatment application (*p* < 0.001). At a further 6-month follow-up check, none of the patients enrolled presented any clinical periodontal damage, such as signs of gingivitis or initial signs of root resorption.

## 4. Discussion

The aim of the present study was to evaluate the influence of LLLT therapy by means of diode laser in accelerating tooth movement and on pain experiencing during OTM. The results showed that, when LLLT was used (test side), it resulted in a significant acceleration in tooth leveling and alignment and in a 29% decrease in the overall treatment time compared to the control side.

The studies currently presented in the literature show the influence of laser-assisted therapy on orthodontic movement on animals, highlighting that, when soft tissue and bone tissues were treated with LLLT, they demonstrated an accelerated process of tissue repair and neoapplication with a consequent increase in the speed of OTM [2,5].

Moreover, it has been shown by several studies that OTM can result in quantitative and qualitative changes in periodontal tissues [5,14,35,36]. These changes in periodontal tissues induced by the orthodontic force are modulated by growth factors, bone metabolism, and some mediators such as interleukins-1ß and some enzymes which, during tooth movement, are increased in response to the orthodontic mechanical stress [36,37]. In this regard, the periodontal benefits of LLLT have been described in previous studies in terms of greater reduction of PD, bleeding sites, and periodontal inflammation, decreasing IL-1b levels in the gingival crevicular fluid (GCF) [38,39], even in the 1st month of implementation.

Based on a previous preliminary finding that LLLT increased the speed of relapse without any retainer [40], it can be hypothesized that LLLT might be potentially important in controlling relapse by accelerating both the degradation and synthesis of collagen of periodontal ligament during OTM.

Immunohistochemical findings showed that LLLT increased Col-I expression and facilitated Metalloproteinases (MMP) gene expression [41]. More specifically, it has been shown that, during the OTM, LLLT stimulated collagen degradation via MMP activation, maintaining balance with collagen synthesis [41,42]. By means of the accelerated collagen repair, the moved teeth establish a new position within the alveolar bone with freshly produced periodontal ligament (PDL) fibers [42].

Furthermore, several studies demonstrated that LLLT therapy is an effective tool for tissue repair, for a reduction in gingival bleeding and of post-surgery bone neo-formation using tissue biostimulation [36,37,43]. The effect of biostimulation induced by LLLT has been shown to determine the increase in wound healing, bone repair and fibroblast growth in the early healing phases after laser application [44] and as stimulation of osteoblast proliferation and intercellular osteoblast exchange during health and disease [45,46].

The effects of tissue biostimulation induced by laser therapy by LLLT at bone neo-apposition level have been shown to be directly proportional to the dose and time of therapy by LLLT [46]. Some studies carried out in vivo and on cell cultures have highlighted a greater regenerative effect, in patients with hematological disorders, by means of LLLT therapy [47,48]. However, the optimal dosing parameters and exposure time to LLLT still need to be precisely determined [49,50].

A study by Luger et al. [51] showed that the use of a dose of 64 J/cm^2^ for 14 days can progressively determine the loss of the force exerted by the laser equal to 3–6% after the first days of application. In our study, the LLLT, that was slightly higher (66.7 J/cm^2^), was applied with the laser tip in contact mode with the gingival tissues (in order to increase the effectiveness of LLLT at the chosen site) at a dose of 66.7 J/cm^2^ with an energy distribution of six different points per treated tooth, however slightly higher than the study by Luger et al. [51]. The reason to apply the LLLT on six points was made because it had been shown that the major application area of the LLLT by means of diode laser may have been one of the greatest factors for biostimulation, resulting in a faster displacement during OTM or implant therapy due to a better distribution of laser energy [52,53,54].

The present study demonstrated also that the test side (diode laser) showed a significant reduction in the average range of dental pain due to orthodontic traction at 3, 7, and 14 days after laser treatment. 

In accordance with the results of the present study, Harazaki et al. [55] found that laser therapy during orthodontic treatment is an optimal tool especially for the reduction of dental pain associated with orthodontic traction. Although the mechanisms responsible for the reduction of pain induced by LLLT during OTM are still unclear [56], it has been shown that LLLT possesses neural and anti-inflammatory periodontal regenerative properties determining the cell differentiation and proliferation useful for pain reduction during OTM [57,58]. Moreover, among the other factors that determine the analgesic effect by LLLT, it has been reported that the stimulation of enzymes aimed at pain-inductive factors also inhibits nervous fiber depolarization (fibers C), the reduction of prostaglandins, and the production of ATP [59,60]. Furthermore, LLLT by means of diode laser has been shown to reduce the pain perception during orthodontic treatment [61,62,63]. More specifically, the use of diode laser, used in a continuous wave, was demonstrated to produce a significant enhancement in the operatory view and in a reduction in pain after tooth movement in the first three days after OTM. This could be useful in some systemic conditions or in patients with periodontitis [64,65,66,67,68].

However, the results of the present study have some limitations to be addressed. One is represented by the study design. The patients were not blinded to treatment and this may have affected the results obtained, especially regarding the pain experience [69,70]. Moreover, the tooth movement was assessed at 2-months follow up and further studies with a longer follow up are needed in order to confirm the preliminary results of the present study.

In conclusion, the results of the present study suggest the positive effects of LLLT by means of diode laser for accelerating OTM and for reducing dental pain linked to OTM. However, further studies are needed on a larger sample to understand better the mechanisms induced by laser therapy during OTM.

## Figures and Tables

**Table 1:** Speed of tooth movement to accomplish space closure in the analyzed teeth.

Group	N°of Analyzed Teeth	Mean ± SD (Days) (95% CI)	*p*-Value
Test side	41	84.35 ± 12.34 (79.3–86.9)	<0.001
Control side	41	97.49 ±11.44 (91.7–102.3)	

**Table 2:** Percentage of leveling and alignment improvement (days). Results are expressed as mean ± SD. A *p* < 0.05 was considered statistically significant. Compared to the control side, the test side showed a significant reduced overall time needed for space closure (*p* < 0.001).

	T1	T2	T3
	Mean ± SD (95% CI)	Mean ± SD (95% CI)	Mean ± SD (95% CI)
Test side	65.36 ± 11.39 (59.9–67.4)	89.42 ± 7.16 (78.2–95.2)	91.1 ± 5.56 (87.3–94.4)
Control side	44.39 ± 15.51 (41.3–48.7)	68.66 ± 15.12 (62.1–71.3)	92.3 6.69 (86.5–95.6)
*p*-value	0.003	<0.001	0.878

## Supplementary Materials

The following are available online at https://www.mdpi.com/1996-1944/12/13/2187/s1, CONSORT 2010 checklist of information to include when reporting a randomized trial.
